# In vitro comparative quality evaluation of different brands of esomeprazole tablets available in selected community pharmacies in Dhaka, Bangladesh

**DOI:** 10.1186/s13104-018-3285-x

**Published:** 2018-03-20

**Authors:** Oby dulla, Sharifa Sultana, Md. Shohag Hosen

**Affiliations:** Department of Pharmacy, Faculty of Allied Health Sciences, Daffodil International University, Dhaka, 1209 Bangladesh

**Keywords:** Esomeprazole, Quality control, Proton pump inhibitor, Disintegration, Pharmacopoeial specifications

## Abstract

**Objective:**

Esomeprazole is the *S*-isomer of omeprazole, used to treat gastro esophageal reflux disease. It is one of the widely manufactured and marketed drugs by many pharmaceutical companies in Bangladesh. The aim of the study is to compare the different physical parameters including hardness, friability, diameter, thickness, disintegration time, dissolution test and assay for quality evaluation and characterization of tablets of five different brands of Bangladeshi pharmaceutical company. The specified compendial method was followed for their evaluation test.

**Results:**

Esomeprazole Mg tablets are enteric coated tablet, there was no disintegration for any brand occurred in 0.1 N HCl after 2 h and all tablets were disintegrated within 19.93 ± 0.04 to 29.05 ± 0.14 min in phosphate buffer (pH 6.8). Weight variation and Hardness were between 1.01 ± 0.29 to 2.01 ± 0.14% and 5.32 ± 0.06 to 7.12 ± 0.12 kgf respectively. Medicine released after 2 h in 0.1 N HCl were varied from 2.55 ± 0.24 to 4.47 ± 0.31% which was less than 10% and in phosphate buffer (pH 6.8) the percentage of medicine release were between 100.9 and 105.9% after 60 min. In case of assay the results of all brands were between 95.28 ± 0.08 and 99.40 ± 0.11%. The obtained results of all parameters were complied with pharmacopoeial limit. So from this study we can conclude that products of esomeprazole available in Bangladeshi pharmaceutical market meet the quality parameter to satisfy therapeutic efficacy.

## Introduction

Esomeprazole is a proton pump inhibitor (PPI), mainly works by preventing the formation of stomach acid through inhibition of the H+/K+ -ATPase in the parietal cells of the stomach [1, 2]. It is used in the treatment of acid-related diseases such as dyspepsia, peptic ulcer disease, gastroesophageal reflux disease and Zollinger-Ellison syndrome. Quality of medicine is an absolute necessity in terms of both therapeutic efficacy and safety of the patients. World Health Organization claimed that the manufacturers must undertake responsibility for the quality of the medicines that they manufacturing [3].

About 300 pharmaceutical companies are manufacturing variety of medicines in Bangladesh. Only 3% of the medicines are imported, the remaining 97% come from local companies [4]. Bangladesh has made agreeable positive developments in the pharmaceutical sector. As a result it is now exporting drugs to many countries across the world including the United States [5]. This sector can develop more and can become one of the major exporting sectors in Bangladesh if these sectors overcome the underlying obstacles like active pharmaceutical ingredient (API) importation [4, 5]. The principal criteria for a quality medicine product are safety, potency, efficacy, and stability [6].

This work will increase awareness among the health practitioners and medicine control authority so that, pharmaceutical manufacturers are forced by them to manufacture quality medicine. This study will also provide a comprehensive knowledge about the weight variation, hardness, disintegration, dissolution, the percentage of the potency of Esomeprazole Mg tablets available in the market and compares these values with the official specifications.

## Main text

### Materials and methods

The specified compendial method was followed for their evaluation test. Five brands of Esomeprazole tablets, manufactured by five different manufacturers of Bangladesh with labelled contents of 20 mg were obtained from retail pharmacy of different areas of Dhaka city. All tablets were of same manufacturing year.

#### Instruments used in the study

Laboratory instruments such as Dissolution Test Apparatus USP (Minhua, RC-8), UV–Visible Spectrophotometer (T60U PG Instruments, England). Electronic Balance (Ohaus CP213 China), Hardness Tester (Monsanto), Friability Tester, and Disintegration Test Apparatus (Aesico, CAT NO 20066B) were used in this study.

#### Reagents used in the study

Sodium hydroxide (CID 14798, Merck, Germany), and 0.1 N HCl (CID 313), potassium dihydrogen phosphate (CID 516951, Scharlu, Spain) and dipotassium hydrogen phosphate (CID: 24450 Scharlu, Spain), distilled water (CID: 962) were used. All the reagents were analytical grade.

#### Collection of samples

A good number of pharmaceutical companies of Bangladesh producing esomeprazole either alone or in combinition forms. There are many brands of Esomeprazole tablets in Bangladesh. Samples were collected from five different batches of every brand which were in same manufacturing year from retail pharmacy of different areas of Dhaka city and collected samples covered top, middle and lower companies ranked by Bangladesh Pharmaceutical Index, 3Q’2011 [7]. The samples were properly checked for their physical appearance, the name of the manufacturer, batch number, manufacturing data, expiration date, manufacturing license number, medicine administration registration number, and the maximum retail price at the time of purchase. The samples were then properly coded for analysis (E01, E02, E03, E04, and E05).

#### Collection of standard

The reference standard of Esomeprazole was obtained from Incepta Pharmaceutical Ltd, Dhaka, Bangladesh as gift sample for research. The purity of the reference standard was 92.11% as esomeprazole which equivalent to 102.5% of esomeprazole magnesium.

### In vitro quality control tests

#### Weight variation test

Twenty tablets from each brand products were weighed individually in a weighing balance. The average weights of the tablet, as well as their percentage deviation, were calculated (Table 1) using following equation [8].

**Table 1:** Evaluation of different quality control parameter of esomeprazole [10, 18, 20, 21]

Sample	Weight variation (%)^a^	Hardness (Kgf)^b^	Disintegration (Min)^c^	Potency (%)^d^
	(Mean ± % RSD)			
E01	1.20 ± 0.23	5.32 ± 0.06	29.05 ± 0.14	95.28 ± 0.08
E02	1.41 ± 0.54	6.71 ± 0.02	26.83 ± 0.09	98.45 ± 0.16
E03	2.01 ± 0.14	5.76 ± 0.14	19.93 ± 0.04	99.40 ± 0.11
E04	1.01 ± 0.29	6.26 ± 0.09	26.28 ± 0.18	98.63 ± 0.13
E05	1.21 ± 0.42	7.12 ± 0.12	28.18 ± 0.02	98.87 ± 0.06
USP specification	NMT ± 5% to ± 7.5%	4–8 kgf	5–30 min	95–105%

^a^20 time replication for each brand

^b^10 time replication for each brand

^c^6 time replication for each brand

^d^3 time replication for each brand

$$ {\text{Weight variation }} = \, \left( {{\text{Iw }}{-}{\text{ Aw}}} \right)/{\text{Aw }} \times { 1}00\% $$

where, Iw = Individual weight of the tablet and Aw = Average weight of the tablet.

#### Hardness test

Hardness indicates the capability of a tablet to withstand mechanical shocks during handling in manufacturing, packaging, and shipping [9]. The hardness of different brands of Esomeprazole tablet was measured by monsanto hardness tester. The test was repeated ten times for each brand. The acceptable limit of hardness of a tablet is 4–8 kgf [10–12]. The hardness of five brands of Esomeprazole was determined and the observed results are shown in Table 1.

#### Disintegration test

6 tablets from each brand were used for the disintegration test in 0.1 N HCl at 37 ± 2 °C for 2 h and then in phosphate buffer (pH 6.8) using a disintegration apparatus. The disintegration time was taken to be the time when no particle remained on the basket [13]. Disintegration is the breakdown process of a tablet into smaller particles and is the first step towards dissolution. Enteric coated are to point out no proof of disintegration once 1 hour in simulated stomach fluid and are to disintegrate in 2 h and the time per the monograph within the intestinal fluid. To be compliance with USP standards, the tablets should disintegrate, and particle must pass through the 3 in. long glass tubes and held against a 10-mesh screen within the time given [14]. The onset of action of a dosage form of a drug depends on the time to be taken by the tablets to unharness the active ingredients into the succus. The tablets ought to be disintegrated within the acceptable time, otherwise, the prescribed course is affected and also the drug might not exert its effect properly. The disintegration time of five brands in phosphate buffer (pH 6.8) of Esomeprazole is shown in Table 1.

### Assay

#### Preparation of standard curve

**Fig. 1:**
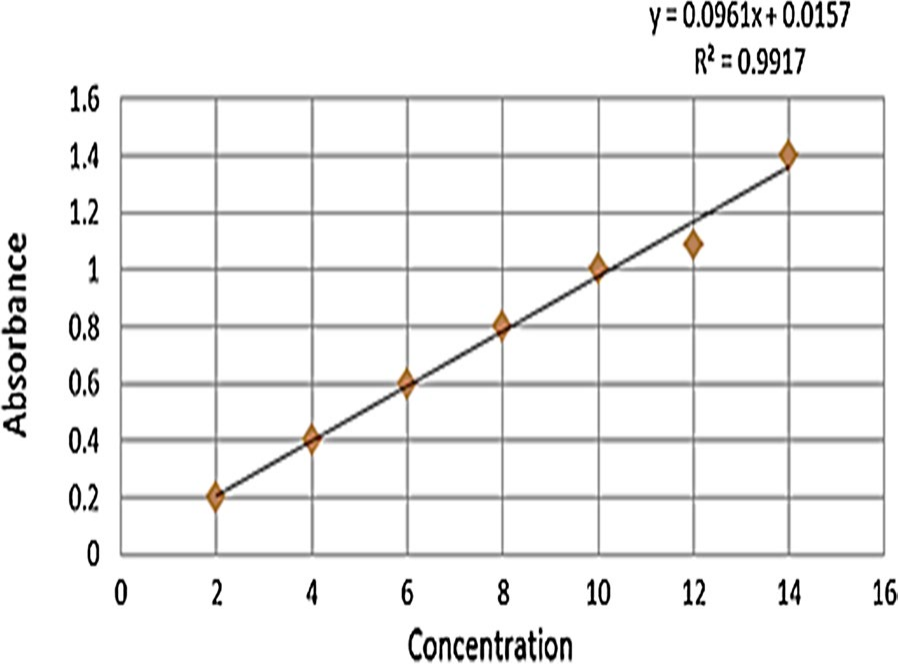
Standard curve of Esomeprazole. A series of the standard solution of Esomeprazole e.g. 2, 4, 8, 10, 12, 16 μg/ml, were taken to measure absorbance at 300 nm against a blank for each solution by UV-spectrophotometer. The measured absorbances were plotted against the respective concentration of the standard solutions which give a straight line with 0.9917 R2 value

Ten milligrams of Esomeprazole Mg was measured by the electronic balance and placed in a 100 ml volumetric flask which was dissolved in phosphate buffer (pH 6.8). The concentration of the solution was attained 100 μg/ml by adding phosphate buffer (pH 6.8) up to 100 ml. 1 ml of a solution was taken from the 100 ml of volumetric flask and phosphate buffer was added up to 10 ml and the concentration was 10 μg/ml. A series of concentrations of the standard solution of Esomeprazole e.g. 2, 4, 8, 10, 12, 16 μg/ml, were taken by above way to measure absorbance at 300 nm against a blank (phosphate buffer) for each solution by UV-spectrophotometer. The measured absorbances were plotted against the respective concentration of the standard solutions which gives a straight line (Fig. 1).

#### Preparation of standard solution

To prepare a standard solution, 20 mg of Esomeprazole was measured by the electronic balance and placed in 100 ml volumetric flask and dissolved in methanol. Then the concentration of the solution was attained 200 μg/ml by adding phosphate buffer (pH 6.8) up to 100 ml. Then 1 ml of a solution was taken from the 100 ml of volumetric flask and diluted to 10 ml with phosphate buffer and absorbance was measured. To get more precise absorbance it was done at least two times.

#### Preparation of assay solution

Ten tablets of each brand of Esomeprazole was weighed and powdered. Esomeprazole magnesium equivalent to 20 mg Esomeprazole was weighed and dissolved in methanol and added phosphate buffer up to about 70 ml. Then the solution was sonicated about 15 min in the sonicator. After cooling the solution, phosphate buffer was added into the volumetric flask up to 100 ml and the solution was filtered. 1 ml of the filtered solution was taken in a test tube and made the volume 10 ml with phosphate buffer. Absorbance was measured at 300 nm using UV spectrophotometer. This was done at least three times for each brand of Esomeprazole.

#### Calculation

The potency was calculated according Beer-Lambert laws [15].

#### Dissolution test

**Fig. 2:**
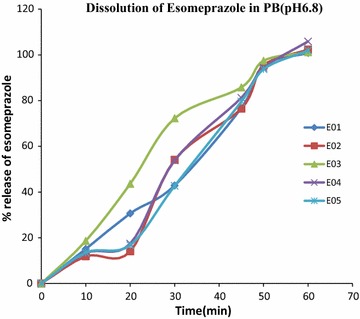
Percent of drug release after 60 min in phosphate buffer (pH 6.8). All the brands meet the specification of the U.S.P standard as they did not release more than 10% drug in 0.1 N HCl after 2 h treatment and release in cases of all brands more than 75% within 45 min in phosphate buffer (pH 6.8)

In-vitro dissolution studies were carried out using dissolution USP apparatus # II. The dissolution medium was 900 ml of 0.1 N HCl and 900 ml of phosphate buffer (pH 6.8), which was maintained at 37 ± 0.5 °C and 75 RPM. In all dissolution experiments, 5 ml of dissolution samples were withdrawn and replaced with the equal volume fresh dissolution medium at regular intervals. Collected dissolution samples were used for determination of released Esomeprazole concentrations by using a UV–VIS spectrophotometer against a blank at 300 nm (Fig. 2).

### Results and discussion

All the brand of Esomeprazole tablets used in this investigation was within their shelf life. The labelled shelf life of all tablets was 3 years from the date of manufacturing. All tablets were subjected to a number of tests in order to assess quality parameters. There are no abnormalities found in the physical appearances of all tablets from different brands.

All tablets obtained from local market were subjected to a number of tests in order to assess quality parameters. The weight variation test is a satisfactory method of determining the medicine content uniformity of tablets and does serve as a pointer to good manufacturing practices (GMP) maintained by the manufacturers as well as the amount of active pharmaceutical ingredient (API) contained in the formulation.

The weight variation for all the tablets used in this study showed compliance within the official specifications [8, 16], as none of the products deviated from the spec. When the weight variation is within the specifications the tablets are thought to contain a uniform active ingredient to give desired therapeutic response but when the weight variation is out of the specification the tablets are thought to contain less or more active ingredient to give an ineffective therapeutic response or toxic effect respectively [17]. It may vary due to result from, poor granulation flow properties, resulting in uneven die fill [18, 19]. It was observed that all the brands meet the USP specification which was between 1.01 ± 0.29 to 2.01 ± 0.14% (Table 1).

Hardness test of material is indicative of its strength. Most important physical feature for assessing tablet is hardness [11]. The acceptable limit of hardness of a tablet is 5–8 kgf. Besides, a force between 4 and 10 kg is also considered to be satisfactory [11, 12, 20, 21]. The hardness of five brands of Esomeprazole was determined and was between 5.32 ± 0.06 to 7.12 ± 0.12 kgf which meet the USP specification (Table 1).

The disintegration time of five brands in phosphate buffer (pH 6.8) of Esomeprazole is shown in Table 1. Before checking the disintegration in phosphate buffer (pH 6.8) they were treated with 0.1 N HCl for 2 h but no disintegration occurs because of its enteric coating. None of the samples exceeded the specification of disintegration time which were between 19.93 ± 0.04 to 29.05 ± 0.14 min. The disintegration time of various brands of Esomeprazole tablet is shown Table 1.

The assay results of all brands were between 95.28 and 99.40% which meet the USP specification for assay test (Table 1).

The dissolution rate of five brands of Esomeprazole tablets was determined. USP specification is NMT 10% in 0.1 N HCl after 2 h and NLT 75% of the labeled amount of Esomeprazole to be dissolved after 45 min in Phosphate buffer (pH 6.8) [10]. Dissolution of esomeprazole tablet of these five brands in 0.1 N HCl were NMT 10% which was 2.55 ± 0.56, 2.64 ± 0.85, 3.88 ± 0.48, 4.47 ± 0.25, 2.94 ± 0.66 respectively whereas the lowest values were 2.55 ± 0.24% and highest value 4.47 ± 0.31% and lowest values were 2.55 ± 0.24% and highest value 4.47 ± 0.31%. Dissolution in Phosphate buffer (pH 6.8) were between 100.9% and 105.9% after 60 min. The five brands of Esomeprazole tablets meet the specifications [22]. The dissolution rate of various brands of Esomeprazole tablet after 60 min is shown in Fig. 2.

### Conclusion

Esomeprazole tablets were analyzed to find their correct quality status. For this purpose, the marketed sample of Esomeprazole tablets was analyzed by using established methods and apparatus. The results of all the parameters obtained from the study comply with the Pharmacopoeial limit. So, on the basis of those results, we can conclude that the products of esomeprazole available in Bangladesh meet the quality parameter to satisfy therapeutic efficacy. All of the brands have proved that they have the quality which meets the official specification. The data reported in this study that the sample produced by lower ranked company also ensure quality product which also met the official requirement. This study can help the Drug Control Authority to get an idea about the quality status of the marketed Esomeprazole preparations in Bangladesh.

### Limitations

For this study small number of company was evaluated to assess the quality. Increasing the number of company will give precise idea about the whole scenario of Esomeprazole tablet in Bangladeshi Pharmaceutical market. Authors also faced difficulties during collection of chemical reagents and the reference standard of Esomeprazole for the analytical tests as that was limited in the research laboratory.

## Abbreviations

USP: United State Pharmacopeia
SD: standard Deviation
BP: British Pharmacopeia
RPM: rotation per minutes
NMT: not more than
NLT: not less than
Kgf: kilogram-force
PB: phosphate buffer
